# COVID‐19 and the boundaries of open science and innovation: Lessons of traceability from genomic data sharing and biosecurity

**DOI:** 10.15252/embr.202051773

**Published:** 2020-10-21

**Authors:** Jusaku Minari, Go Yoshizawa, Nariyoshi Shinomiya

**Affiliations:** ^1^ Uehiro Research Division for iPS Cell Ethics Center for iPS Cell Research and Application (CiRA) Kyoto University Kyoto Japan; ^2^ Visiting Researcher Innovation System Research Center Kwansei Gakuin University Hyogo Japan; ^3^ National Defense Medical College Research Institute Saitama Japan

**Keywords:** S&S: Economics & Business, S&S: Ethics

## Abstract

The COVID‐19 crisis has further highlighted the challenges for open science and data sharing in biomedical research and the need for more traceability and transparency.

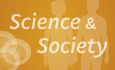

Many aspects of society are subject to increasing digitalisation and science has been at the forefront. The wide usage of digital technologies in research inspired the concept of Open Science that is increasingly playing an essential role in advancing research and technology. Open Science, enabled by digital communication technologies, aims to make publicly funded scientific research available to any scientists or other interested party by freely sharing results, data, methods, reagents, technologies and so on. Major funding initiatives and agencies such as EU's Horizon Europe Programme or the NIH are supporting Open Science through specific policies that mandate open access to published research and sharing of data and resources.

However, these mandates and policies can clash with privacy, data protection and security. Privacy and data protection legislation, notably the EU General Data Protection Regulation (GDPR) reign supreme over data sharing for human‐related biomedical research. Additionally, the free sharing of data on pathogens and infectious diseases raises issues of biosecurity: the risk that the information could be abused for nefarious purposes. These issues have been further highlighted by the ongoing COVID‐19 crisis as it challenges scholarly publishing to rapidly make important information about the pandemic publicly available. In fact, COVID‐19 holds important lessons for more feasible and sustainable approaches towards “openness” than mere mandates and policies.

## Visualisation of genomic data sharing

Genomic data sharing in precompetitive research has been long established and become a poster child of open science. Over years, it has developed various approaches and tools to enable and encourage sharing: data‐sharing policies; efficient data access systems; large and reliable public databases; ethical and legal frameworks for sharing personal and patient data; and international coordination between scientists, publishers and public funding agencies. These approaches aim to manage the tension between data protection, protecting research participants’ privacy, appreciating their voluntary participation, and facilitating data‐driven research and innovation.

…COVID‐19 holds important lessons for more feasible and sustainable approaches towards “openness” than mere mandates and policies.

However, there is one important caveat here: the lack of a shared sense of purpose between research participants and data users, especially end users. The aforementioned EU GDPR and other legislation primarily focus on the identifiability of data to protect privacy and private data (Shabani & Marelli, 2019), and thereby reasonably require anonymisation and safeguards such as pseudonymisation to health‐related and genomic data. Yet, the overemphasis of identifiability undervalues the origin, process and utility of the data and prevents a sense of sharing in the spirit of Open Science between donors and users. Remote and faceless data collection, storage and analysis enterprises from high‐throughput sequencing to large databanks and cloud computing technologies, further drive a wedge between patients and research participants, and scientists and other users. While the first group has only limited control over and understanding of how, by whom and for which purpose their data are stored, processed and used, the latter often see genomic data simply as a resource to be mined for research.

Both scientists and participants would benefit from a more robust genomic data management and control system. A possible solution could visualise data‐sharing practices to build a greater awareness among both data users and (potential and existing) research participants of how data is being used, and to encourage more data sharing. This could take the form of web tools for large‐scale international research projects and public databases that show the numbers of research participants at various scales such as the level of institutions, regions and nations – adjusting scales based on data sensitivity – as well as clarify the process of data transformation and data curation. While this still prevents identifiability, such visualisation would give donors and users a tool to trace the origin of data, its flow, usage and governance, akin to the idea of Biobankland (Kauffmann & Cambon‐Thomsen, 2008) and Bioresource Research Impact Factor (Cambon‐Thomsen *et al*, 2011). To achieve more informed decision‐making and trust, in particular among research participants, traceability and utility are important tool that would be complementary to data protection under the GDPR and similar legislation.

A possible solution could visualise data‐sharing practices to build a greater awareness among both data users and (potential and existing) research participants of how data are being used, and to encourage more data sharing.

## Dual‐use research of concern and biosecurity review

Second, there is now a risk that “openness” on public health issues in scholarly publishing could be abused. Even before the COVID‐19 pandemic, some infectious diseases, research on and publications about pathogen including influenza A virus subtype H5N1 (Herfst *et al*, 2012; Imai *et al*, 2012) or horsepox virus (HPXV) (Noyce *et al*, 2018), raised issues about the justifiability of such studies in light of dual‐use research of concern (DURC) and its risk‐benefit analysis.

Biosecurity reviews do not provide an adequate or effective solution for preventing data misuse and abuse of published virus genome sequences. Such reviews merely discuss whether the risk of abuse is sufficiently high and might recommend that the article should not be published, but journals or reviewers hardly ever reject submissions on the grounds of biosafety or biosecurity (Patrone *et al*, 2012). A possible remedy would again be ensuring justifiability and traceability to a reasonable extent, and more journals should invite interdisciplinary experts and stakeholders to assess DURC and publish reviewers’ comments along with the publication as some publishers already do.

## Sharing knowledge on COVID‐19

COVID‐19 has raised various urgent question about the origin, the transmission and the pathogenicity of the SARS‐CoV‐2 virus. How did the genetic recombination occur in the natural world? What are the mechanisms of infection? Which drugs could be used to prevent the severe side effects and long‐term damage of the infection? Several sequence and structural studies have suggested that the virus originates from a natural recombination/selection between bat coronavirus and pangolin coronavirus, before a zoonotic transfer to humans (Andersen *et al*, 2020; Shang *et al*, 2020), however, the home ranges and habitats of these animals rarely overlap. Clinical evidence suggests that the virus can spread to other organs and tissues beyond the respiratory system and cause long‐term damage. And clinicians around the world have been frantically searching for drugs and therapies to prevent the severe manifestations of SARS‐CoV‐2 infection that cause death and long‐term tissue damage. This situation has necessitated a rapid and open form of COVID‐19‐related knowledge‐sharing with the global research community.

Biosecurity reviews do not provide an adequate or effective solution for preventing data misuse and abuse of published virus genome sequences.

As such, the COVID‐19 crisis underlines two double‐edged concepts related to openness in scholarly publishing. One is the rapidly increasing popularity of online publication of preprints via MedRxiv and BioRxiv. Preprint publishing has indeed greatly helped researchers to quickly and efficiently spread, share and utilise COVID‐19‐related knowledge without the inevitable delay by editorial and peer review. However, preprints, by their very nature, are not checked and validated for quality and conclusiveness by peer review. In this regard, preprints and other kind of not peer‐reviewed reports should not be regarded as conclusive evidence, but rather as work in progress. Moreover, preprints are not scrutinised for biosecurity issues, which could create an additional risk for nefarious or criminal abuse of biological information – though, in fairness, most journals do not always carefully look for these issues either. In the longer term, we need to analyse the advantages and drawbacks of preprint publishing and its scientific, responsive and inclusive aspects. While preprints have fulfilled an important role in rapidly disseminating information, quality and other issues could be addressed by developing open, transparent post‐publication review systems with registered reviewers.

… the COVID‐19 crisis underlines two double‐edged concepts related to openness in scholarly publishing.

Of greater concern though are problems with peer review and publishing in high‐impact journals that have become more apparent during the COVID‐19 pandemic. Two articles about possible treatments for SARS‐Cov2 infection in *The Lancet* and *the New England Journal of Medicine* were quickly retracted in response to widespread criticism of their methods and data resources (Gannon, 2020). However, the failure by the current review system to spot the weaknesses of these articles can be, at least in part, owing to the increased pressure on journals to quickly review and publish clinically relevant data on the pandemic. A possible solution to address such blunders with potentially grave consequences for public health in emergency situations could be a post‐publication review system for relevant articles. More generally, Open Science could again help to alleviate the ongoing problems with and serious challenges for the peer review and publishing system by encouraging transparency, accessibility and accountability.

In terms of genomic data management, traceability is probably a more appropriate openness‐related function than transparency and accessibility, both of which apply only to the user interface not the system itself. If the data are not traceable, it raises doubts not only about the quality of the data itself but also whether research participants gave adequate informed consent. As shown in the case of the retracted articles from two high‐impact journals, such a lack of traceability can affect publishing particularly in emergency situations.

While conventional policies and systems for data sharing and scholarly publishing are being challenged and new Open Science policies are being developed, traceability should be a key function for guaranteeing socially responsible and robust policies. Full access to the available data and the ability to trace it back to its origins assure data quality and processing legitimacy. Moreover, traceability would be important for other agencies and organisations – funding agencies, database managers, institutional review boards and so on – for undertaking systematic reviews, data curation or process oversights. Thus, the term “openness” means much more than just open access to published data but must include all aspects of data generation, analysis and dissemination along with other organisations and agencies than just research groups and publishers. The COVID‐19 crisis has highlighted the challenges and shortfalls of the current notions of openness and it should serve as an impetus to further advance towards real Open Science.

## Conflict of interest

The authors declare that they have no conflict of interest.
